# A review of skin immune processes in acne

**DOI:** 10.3389/fimmu.2023.1324930

**Published:** 2023-12-15

**Authors:** Zhongcai Jin, Yujun Song, Li He

**Affiliations:** Skin Health Research Center, Yunnan Characteristic Plant Extraction Laboratory, Kunming, Yunnan, China

**Keywords:** acne vulgaris, immune response, microorganism, lipid mediators, neuropeptides, single-cell analysis

## Abstract

Acne vulgaris is one of the most prevalent skin conditions, affecting almost all teenagers worldwide. Multiple factors, including the excessive production of sebum, dysbiosis of the skin microbiome, disruption of keratinization within hair follicles, and local inflammation, are believed to trigger or aggravate acne. Immune activity plays a crucial role in the pathogenesis of acne. Recent research has improved our understanding of the immunostimulatory functions of microorganisms, lipid mediators, and neuropeptides. Additionally, significant advances have been made in elucidating the intricate mechanisms through which cutaneous innate and adaptive immune cells perceive and transmit stimulatory signals and initiate immune responses. However, our understanding of precise temporal and spatial patterns of immune activity throughout various stages of acne development remains limited. This review provides a comprehensive overview of the current knowledge concerning the immune processes involved in the initiation and progression of acne. Furthermore, we highlight the significance of detailed spatiotemporal analyses, including analyses of temporal dynamics of immune cell populations as well as single-cell and spatial RNA sequencing, for the development of targeted therapeutic and prevention strategies.

## 1 Introduction

Acne vulgaris is a dermatological condition that predominantly affects approximately 85% individuals in adolescence and early adulthood (1, 2). Acne tends to occur in regions characterized by a high concentration of sebaceous glands, such as facial and upper back regions (3). Pilosebaceous units (PSUs), composed of sebaceous glands and hair follicles, are the fundamental structures affected in acne lesions. In typical PSUs, the production and secretion of sebum (a mixture of lipids) are primarily carried out by the sebaceous glands. Secreted sebum travels through the sebaceous duct and enters the lumen of the hair follicle channel, where it coats the keratinocyte wall. The commensal microbiota within the hair follicle possesses the ability to metabolize specific lipid species into free acids, resulting in an environment with a low pH, hindering the colonization or proliferation of harmful microorganisms.

Acne vulgaris manifests when the harmonious equilibrium within the PSU is disturbed. Considering the hair follicle duct as a conduit, hypercornification of the hair infundibulum combined with excess sebum, microorganisms, and keratin squamae can result in the development of microcomedones. These microcomedones subsequently develop into either white or black comedones (4), causing the obstruction of the hair follicle ducts.

Comedones coupled with the excessive production of sebum establish a relatively anaerobic environment, which facilitates the proliferation of specific species of microorganisms, ultimately resulting in dysbiosis of the skin microflora. The altered composition of microorganisms in the PSUs along with the virulence factors they release in conjunction with the enlarged comedones exert pressure on the wall of hair follicles, leading to their compression and subsequent rupture. This process ultimately compromises the structural integrity of the skin barrier within the hair follicles.

Subsequently, invading pathogens, their secreted virulence factors, and degraded sebum penetrate the dermis and activate immune cells, resulting in an intensified inflammatory response. This process results in the development of inflammatory lesions, including papules and pustules. In patients with severe acne, papules and pustules can lead to the development of nodules or cysts. Owing to the destruction of the dermis or hypodermis, certain lesions pose challenges in terms of restoration, ultimately leading to scar formation.

Prior studies have established that the immune system plays a critical role in all stages of acne development. This review provides a comprehensive overview of the immune processes involved in acne development, including a summary of the stimulators that activate the immune response, the mechanisms involved in both innate and adaptive immune responses, and the sequence of infiltrated immune cells in different types of acne lesions.

## 2 Stimulators triggering immune response

Substances that interfere with the regular functioning of PSUs generally stimulate innate immune responses. At present, these substances can be categorized into two distinct types: (1) exogenous substances originating from the external environment, including a diverse range of microorganisms and their virulent metabolites, and (2) autoantigens generated by the host, such as specific lipid mediators from sebum and blood and neuropeptides secreted by neuroendocrine cells. Stimulators with experimental evidence using human cells or tissues are listed in **Table 1**.

**Table 1:** Experimentally evidenced stimulators and receptors of immune responses in acne development.

Stimulators	Receptors	Response cell/tissue	References
Microorganisms-related			
*C. acnes*	TLR2, TLR4, CD14, PAR-2	KCs	(5–8)
		PBMCs	(9–11)
		Monocytes	(12)
		CD3^+^ T cells	(13)
		CD4^+^CD45R T cells	(9, 13)
		Explants	(14, 15)
		Sebocytes	(16, 17)
		THP-1 cells	(12)
*Malassezia species*		PBMCs	(18)
		KCs	(19)
		Acne lesions	(20)
*Staphylococcus species*	TLR2	KCs	(21)
		PBMCs	(11)
		Acne lesions	(22, 23)
Porphyrin III		KCs	(24)
		Acne lesions	(25)
CAMP1	TLR2	KCs	(26, 27)
		Acne lesions	(28)
Extracellular vesicles	TLR2	KCs	(29)
		THP-1 cells	(29)
HSP60		KCs	(30)
SCFAs		Monocytes	(31)
		KCs	(31)
Lipase		Acne lesions	(20, 22, 32)
Lipoprotein	TLR2	Monocytes	(33)
		KCs	(34)
Enterotoxin B		PBMCs	(9)
Lipid mediators			
Oleic acid	CD36	Sebocytes	(35)
Lauric acid	CD36	Sebocytes	(35)
Palmitic acid	CD36	Sebocytes	(35)
Squalene		KCs	(36)
		TREM2^+^ macrophages	(37)
Neuropeptides			
Substance P		Sebocytes	(38, 39)
CRH	CRH-R	Sebocytes	(5, 40)
α-MSH	MC-1R	Sebocytes	(40)

CAMP, Christie-Atkins-Munch-Peterson; HSP, heat shock protein; SCFAs, short-chain fatty acids; CRH, Corticotropin-releasing hormone; TLR, Toll-like receptor; PAR-2, proteinase-activated receptor-2; CRH-R, corticotropin-releasing hormone receptor; α-MSH, alpha-melanocyte stimulating hormone; MC-1R, melanocortin-1 receptor; KCs, keratinocytes; PBMCs, peripheral blood mononuclear cells.

### 2.1 Skin microbiome

The skin microbiome consists of bacteria, viruses, fungi, and archaea that reside in or temporarily inhabit the skin or its appendages (41). The human skin provides diverse microhabitats (differing in thickness, moistness, gland and hair follicle density, and other parameters) for various microbial communities. The most frequently isolated microorganisms in hair follicles are *Cutibacterium acnes* and *Staphylococcus epidermidis* (42).

The capacity of *C. acnes* to elicit an immune response has been evaluated extensively (14). Previous *in vivo* and *in vitro* studies have demonstrated that certain strains of *C. acnes* as well as their toxic metabolites and cell wall components, such as peptidoglycan (PGN), lipoteichoic acid (LTA), and short-chain fatty acids (SCFAs) produced under lipid-rich hypoxic conditions, can induce a significant increase in cytokine expression in cultured keratinocytes (12, 31), sebocytes (16, 17), peripheral blood mononuclear cells (PBMCs) (9, 29), and monocytes (12, 31). The activation of the skin immune system in response to *C. acnes* has also been demonstrated *in vivo*. For instance, Ashbee et al. demonstrated that the levels of IgG1 and IgG3 antibodies targeting *C. acnes* were higher in individuals with severe acne than in those with normal skin, whereas IgG2 specific to *C. acnes* was higher in patients with moderate-to-severe acne than in those with mild acne (43). These *in vivo* results suggest that *C. acnes* plays a progressive role in acne of varying severity.

Despite evidence that *C. acnes* contributes to the development of acne, a consistent difference in the relative abundance of this bacterium between individuals with and without acne has not been detected (44–46). There is a widely accepted consensus that the dysbiosis of *C. acnes* at the strain level, the presence of virulent genetic elements, and altered transcriptional activity provide a more comprehensive explanation for the observed functional disparities between individuals with healthy skin and those with acne. This viewpoint is supported by several studies (15, 25, 42, 46–51).

In addition to the extensively studied *C. acnes*, other microorganisms, such as the most abundant skin commensal fungal genus *Malassezia* and species of *Staphylococcus*, are associated with acne (44, 52, 53). The potential impact of *Malassezia* on the pathogenesis of acne is supported by its positive response to antifungal agents in cases of refractory acne (54), its increased abundance in young individuals with acne (20, 55), increased levels of secreted lipases and stimulation of immune responses in PBMCs and keratinocytes (18, 48, 56).

There is evidence for associations between *Staphylococcus* species and acne. For example, they are highly abundant on the surfaces of comedones, papules, and pustules (45). Furthermore, the occurrence of *S. epidermidis* is higher in patients with acne than in heathy controls (57, 58). The NF-κB pathway is activated in keratinocytes upon treatment with *S. epidermidis* (21) and the mitogen-activated protein kinase (MAPK) is activated by *S. aureus* (59). However, Xia et al. claimed that LTA generated by *S. epidermidis* could inhibit the proliferation of *C. acnes* and reduce the protein expression of toll-like receptor (TLR)-2 in keratinocytes (60). Further studies are required to elucidate the functions of *S. epidermidis* in acne development and resolve inconsistencies.

Given that the immune responses of cultured cells to microorganisms or their secreted virulence factors depend on direct physical contact *in vitro*, it is crucial to determine whether pathogens co-localize with the same cells *in vivo*. To address this issue, Alexeyev et al. used fluorescent *in situ* hybridisation, immunofluorescence microscopy, and immunohistochemistry. They observed that within the hair follicle, both microcolonies and biofilms of *C. acnes* were present on the follicular wall, indicating that there is a direct interaction between *C. acnes* and keratinocytes (32). Using quantitative PCR, Gram staining, immunofluorescence microscopy, and 16S ribosomal RNA sequencing, Nakatasuji et al. identified acne-associated *C. acnes* and *S. epidermidis* within dermal tissues. This finding provides evidence for direct physical interactions between bacteria and different cells in the dermal layer (61). Further studies are necessary to obtain more accurate observations of the direct interactions between particular microbial species as well as secreted virulence factors and skin cells in different acne lesions.

### 2.2 Lipid mediators

Lipids that function as immune-stimulating substances in acne mainly originate from sebum (62). Under typical circumstances, sebum contributes to the defense of the skin against pathogens and maintenance of moisture. However, several studies have shown that changes in the composition of lipid species as well as the oxidant/antioxidant and saturated/unsaturated ratios may convert lipids into immune stimulators during the development of acne (35, 36, 62–66). For instance, sebum free fatty acids, such as lauric acid, palmitic acid, and oleic acid, can enhance the innate immune defense of sebocytes by upregulating a human antimicrobial peptide (AMP), human β-defensin (hBD)-2 (35); Additionally, peroxidated squalene can upregulate the expression levels of inflammatory mediators, such as NF-κB, peroxisome proliferator-activated receptors (PPAR)α, and IL-6 (36); Furthermore, quantities of sebum oxidation-induced lipid peroxide (LPO) and interleukin (IL)-1α are higher in the inflammatory comedones than in non-inflammatory comedones, suggesting that a certain amount of LPO may be involved in inflammatory changes in early acne lesions (67).

Various lipid species come from not only sebum but also other skin surface lipids (62) and serum (68). Although several studies have reported characteristic differences of these lipids between patients with acne and healthy controls (66, 68–72), further research is needed to understand the mechanisms linking these lipids to immune activity.

### 2.3 Neuropeptides

Acne vulgaris is often exacerbated in individuals with mental stress or endocrine dyscrasia (58, 73, 74). This highlights the association between acne and the neuroendocrine system. As a crucial component of the peripheral neuroendocrine system, human skin not only acts as a recipient of signals from various neuropeptides secreted by the central nervous system and transported via the bloodstream but also produces neuropeptides that modulate skin cells via paracrine, juxtracrine, autocrine, and intracrine pathways (75–77).

Obligate immune cells, such as mast cells, Langerhans cells, and macrophages, as well as nonobligate immune cells, such as sebocytes, melanocytes, endothelial cells, and keratinocytes, have been identified as targets of neuropeptides in the cutaneous immune system (75, 78). For example, calcitonin gene-related peptide (CGRP) secreted by skin sensory nerve fibers stimulate the adhesion of leukocytes and monocytes to endothelial cells as well as the release of proinflammatory mediators, such as tumor necrosis factor (TNF)-α and IL-8, from mast cells (79).

However, limited studies have characterized the direct effect of neuropeptides on the immune response in acne. Corticotropin-releasing hormone (CRH), a neuropeptide, shows significantly stronger expression in sebaceous gland cells of acne-affected skin than in non-affected skin (40). It can be secreted by the hypothalamic-pituitary-adrenal axis, keratinocytes, melanocytes, dermal fibroblasts, or endothelial cells and targets one of its receptors, corticotropin-releasing hormone receptor 2 (CRH-R2), to stimulate the release of IL-6 and IL-8 in SZ95 sebocytes (5). Substance P (SP), another neuropeptide, is present at higher concentrations in the nerve fibers around sebaceous glands in patients with acne than in healthy controls (38). Cultured sebocytes treated with SP exhibit increased secretion of proinflammatory cytokines, including IL-6, IL-1, and TNF-α (39).

Further studies are needed to determine the secretory patterns of other neuropeptides in patients with acne and their potential to initiate an immune response.

## 3 Innate immune response

In response to aforementioned immunostimulators, immune-related cells in the skin show alterations in proliferation and/or differentiation as well as in signaling and/or metabolic pathways. This response results in the production of defensin substances or secondary signaling molecules that activate the immune systems.

**Figure 1:**
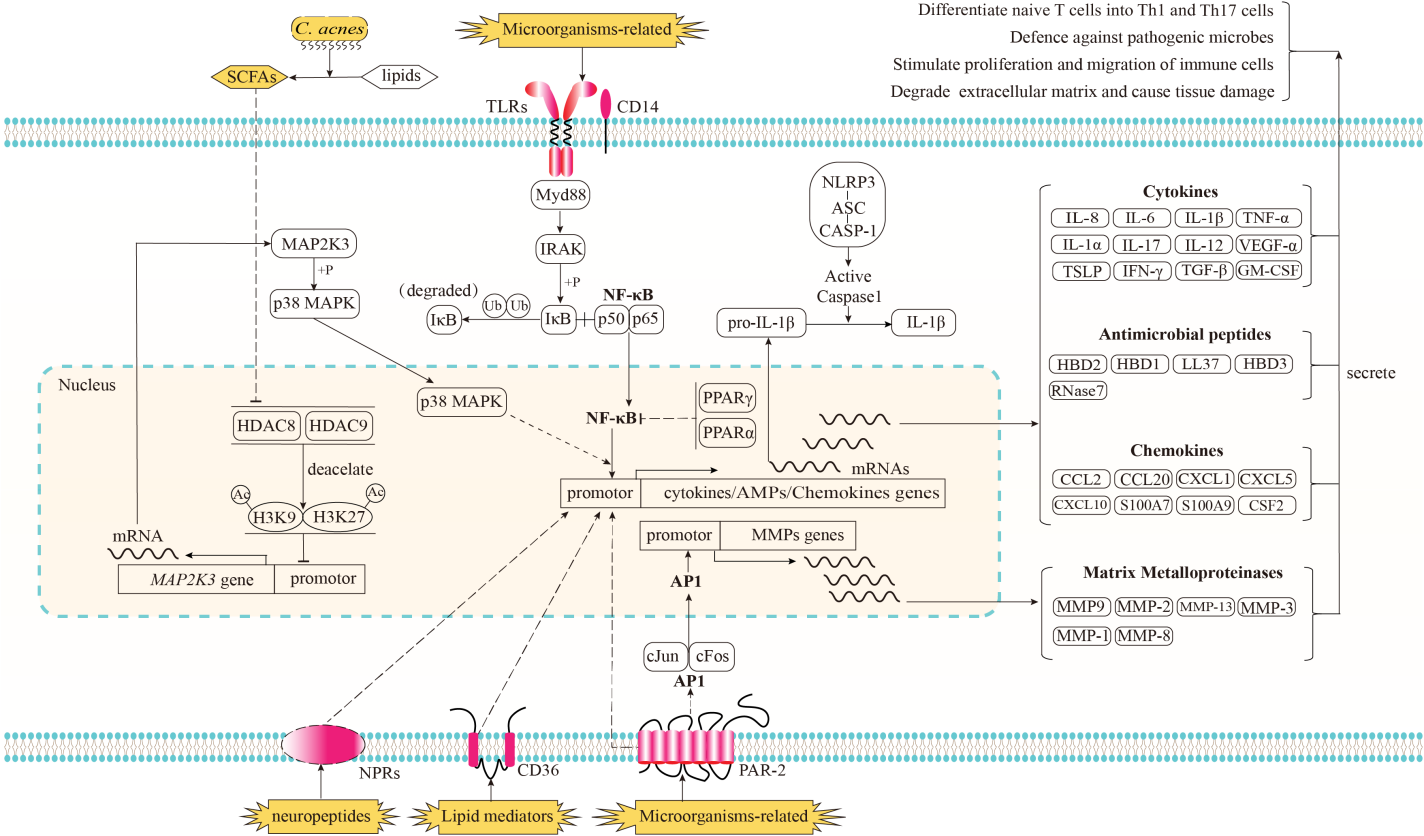
Recognition of immune stimulators and the signaling pathways implicated in the innate immune response of acne and its subsequent biological effects. Microorganisms-related stimulators can be detected by Toll-like receptors (TLRs) in conjunction with CD14 and proteinase-activated receptor-2 (PAR2). The activation of TLRs triggers the downstream NF-κB signaling pathway, resulting in the translocation of NF-κB into the nucleus and the upregulation of genes encoding cytokines, chemokines, and antimicrobial peptides (AMPs); The activation of PAR2 has been shown to elicit the transcriptional upregulation of genes encoding cytokines, chemokines, and AMPs via an unidentified pathway. Additionally, PAR2 activation triggers the downstream signaling pathway of activator protein-1 (AP-1), resulting in the translocation of AP-1 into the nucleus and an enhanced transcriptional expression of matrix metalloproteinases (MMPs). As potent anti-inflammatory factors, the nuclear receptors, peroxisome proliferator-activated receptors (PPARs) PPARα and PPARγ have the ability to inhibit the activation of NF-κB. When cultivated in an environment rich in lipids, the anaerobic fermentation of *C. acnes* can produce short chain free fatty acids (SCFAs). Certain species of SCFAs have the ability to inhibit the deacetylation function of histone deacetylase (HDAC) 8/9. The inhibition of HDAC8/9 consequently results in an amplification of the acetylation process on histone residues H3K9 and H3K27, which marker the promoter region of *MAP2K3*. This, in turn, leads to an enhanced transcription of *MAP2K3*. The heightened expression level of MAP2K3 then triggers the phosphorylation of p38 MAPK, ultimately resulting in the activation of p38 MAPK and an increase in the expression of genes responsible for cytokines and chemokines. Lipid mediators produced by sebaceous glands, such as certain species of free fatty acids (FFAs), have the potential to be identified by the lipid translocator CD36, while neuropeptide stimulators are believed to be recognized by their corresponding neuropeptide receptors (NPRs). Both of these mediators have the ability to enhance the expression of genes involved in immune responses, although the specific signaling pathways through which these receptors and immune response genes operate remain unclear. Prior to being released in an active state into the extracellular region, the inactive form of proinflammatory cytokines, such as pro-IL-1β, necessitates proteolytic processing. This processing is facilitated through the activation of the NLRP3 inflammasome complex. Subsequently, proteins of cytokines, AMPs, chemokines, and MMPs are secreted into the extracellular regions in order to regulate the functioning of neighboring cells, thereby resulting in a cascade of subsequent effects.

The rapid and nonspecific immune response for the prevention of the rapid spread of antigens is referred to as innate immunity. In typical skin, a relatively low pH and low oxygen microenvironment, epidermal keratinocytes in the hair follicles serve as the initial barrier against a multitude of harmful microorganisms and external factors. When the integrity of this barrier is compromised due to imbalances in physiological activity or excessive stimulation from external factors, antigens can penetrate the epidermis and reach the dermis, thereby triggering a more intense and uncontrolled inflammatory response in the deeper layers of the skin. The components of the cutaneous innate immune system, including skin cells (follicular keratinocytes, sebocytes, melanocytes and Langerhans cells), haematopoietic cells, and soluble factors (e.g., cytokines and AMPs) have been comprehensively documented by Dreno et al. (80). In this section, we illustrate the intricate processes by which immune-related cells in acne identify immune stimulators, transmit signals within cells, and generate responses. A schematic of these immune processes is shown in **Figure 1**.

### 3.1 Recognition

TLRs, especially TLR2 and TLR4 in conjunction with CD14, are major receptors involved in the recognition of microorganisms or microbial-derived factors, such as PGN and LTA. These transmembrane proteins have been discovered in culture systems and within skin tissues. They are expressed in various skin cells, such as keratinocytes (81, 82) and sebocytes (16, 83), and in haematopoietic cells, such as PBMCs (10) and monocytic (12, 84). Their expression levels are positively regulated by *C. acnes* (81) and negatively regulated by retinoids (10). In addition to TLRs, proteinase-activated receptor-2 (PAR-2) is directly stimulated by proteases produced by *C. acnes*. Lee et al. found that PAR-2 levels are higher in keratinocytes and sebaceous glands of acne lesions than in non-lesional skin. Lee et al. found that PAR-2 levels are higher in keratinocytes and sebaceous glands of acne lesions than in non-lesional skin (85, 86). *In vitro* experiments using cultured keratinocytes and sebocytes further demonstrated the role of PAR-2 in mediating innate immunity and sebaceous lipogenesis.

Few studies have evaluated signal-receiving elements responsible for the recognition of various lipid mediators. Only free fatty acids, including lauric acid, palmitic acid, and oleic acid, have been shown to be transported by the transmembrane lipid translocator CD36 in cultured SZ95 sebocytes (35).

Neuropeptides stimulate immune responses through recognition by their corresponding neuropeptide receptors (40, 79, 87). However, studies of the roles of neuropeptide-mediated immune responses in acne are limited.

### 3.2 Signal transduction

When TL2 or TL4 binds to antigens with the assistance of its co-receptor, CD14, its cytoplasmic TIR domains interact with the TIR domain of Myd88, an adaptor downstream of TLRs and IL-1 receptors. The death domain of Myd88 interacts with IL-1R-associated kinase (IRAK) family kinases via homotypic protein–protein interactions (88). Activated IRAK stimulates the NF-κB signaling pathway. In humans, NF-κB is a transcription factor in a complex consisting of p50 (NF-κB1) and p65 (rel-A) subunits (89). In normal conditions, NF-κB is sequestered in the cytoplasmic region by binding to the inhibitor of κB (IκB) in the cytoplasmic region. After receiving the upstream inflammatory signals, IκB kinase (IKK) is activated to phosphorylate IκB, and the phosphorylated IκB will undergo ubiquitylation and proteasomal degradation, resulting in the translocation of NF-κB members into the nucleus (90). The nuclear translocation of NF-κB positively regulates the mRNA expression of proinflammatory cytokines.

PAR-2 enables the activation of activator protein-1 (AP-1). Once activated, AP-1 is translocated into the nucleus and promotes the transcription of matrix metalloproteinases (MMPs) (85). However, the downstream effectors that mediate the PAR-2 pathway and stimulate cytokines and AMPs in acne have not been determined.

Before they are released into the extracellular region in an active form, the inactive form of proinflammatory cytokines, like pro-IL-1β, requires proteolytic processing. This process is dependent on the proteolytic activity of caspase-1. The inactive form of pro-caspase-1 is a part of the nucleotide-binding oligomerization domain, leucine-rich repeat, and pyrin domain-containing protein (NLRP) inflammasome complex. Several NLRPs have been characterized according to the types of pathogen-associated molecular patterns (PAMPs) and damage-associated molecular patterns (DAMPs) they are activated by (6). In acne vulgaris, NLRP3 contributes to the recognition of *C. acnes* in human monocytes, and its activation by *C. acnes* requires ROS, K^+^ efflux, phagocytosis, and lysosomal destabilization (12). The activation of NLRP3 leads to proteolytic cleavage of the caspase recruitment domain (CARD) of pro-caspase-1, resulting in active caspase-1 and subsequent proteolysis of pro-IL-1β into mature and secreted active IL-1β (91).

PPARs act as anti-inflammatory factors. Dozsa et al. observed that patients with acne have lower expression levels of PPARγ and its target genes in sebocytes than those in healthy controls (92). Ottaviani et al. found that in cultured keratinocytes, peroxidated squalene could induce the secretion of the proinflammatory cytokine IL-6 through the activation of NF-κB. In this inflammatory environment, the PPARα expression level is increased, supporting the feedback reaction of PPARs to reduce inflammation via the inhibition of the NF-κB pathway (36).

Recently, histone deacetylases (HDACs) were identified as negative regulators inhibiting TLR-induced cytokine expression in keratinocytes. This regulation is crucial for maintaining immune tolerance under normal microbial conditions (31). Under hypoxic growth condition with lipid sources, *C. acnes* utilizes lipids to produce SCFAs, which in turn inhibit the activity of HDAC8 and HDAC9 (31). The inhibition of HADC8/9 increases the acetylation of histone residues H3K9 and H3K27, which mark the promoter region of *MAP2K3*. This increased level of acetylation opens the chromatin in *MAP2K3* and activates the facilitates chromatin transcription (FACT) complex, ultimately increasing the transcription of *MAP2K3*. The heightened expression of MAP2K3 is responsible for the phosphorylation of p38 MAPK and subsequent increased expression of IL-6, IL-8, TNF-α, thymic stromal lymphopoietin (TSLP), chemokine (C-C motif) ligand 5, and IFN-β (31, 93). IFN-β activates cutaneous immunity by promoting dendritic cell (DC) maturation and subsequent T cell proliferation (93).

### 3.3 Production of immune-related factors

After recognizing stimulators and modulating intracellular signaling pathways, skin immune cells produce and secrete immune-related soluble factors, including AMPs, cytokines, chemokines, and MMPs. AMPs are 12–50 amino acid, cationic, and amphiphilic peptides. In human skin, the best-characterized AMPs are cathelicidins and β-defensins (35, 94). They are produced in human keratinocytes and sebocytes in response to stimulators, like *C. acnes*, PGN, LPS, and *Malessezia furfur* (19, 95). AMPs directly inhibit *C. acnes* proliferation and immunomodulation by inducing angiogenesis and cytokine release (94).

Cytokines are regulators produced by host cells in response to infections and immune responses. IL-6, IL-8, IL-1β, and TNF-α are the most well-studied cytokines in acne research. IL-6 and IL-8 are secreted by monocytes (12), keratinocytes (31), PBMCs (29), and sebocytes (39) when stimulated by *C. acnes* or SP. Elevated expression levels of IL-6 and IL-8 in acne lesions also have been reported (33, 96). IL-1β expression is induced in PBMCs (10, 29), monocytes (12), and keratinocyte (31) when stimulated by *C. acnes* and SCFAs produced by *C. acnes*. IL-1 receptors (IL-1R) expressed on the membrane surface can transduce IL-1β signals into intracellular signals to activate NF-κB and AP-1 signaling pathways (89). These activated signals in effector cells promote cytokine production. TNF-α could be stimulated by *C. acnes* in cultured Th1 cells (29), infundibular keratinocyte (12), sebocytes (39), and monocytes (12) when stimulated by *C.acnes* or SP. Secreted TNF-α interacts with TNF-α receptors, stimulating the expression of vascular intercellular adhesion molecule 1 (ICAM-1) and increasing the activity of NF-κB and AP-1 (89). In addition to the aforementioned well-studied cytokines, a recent study has discovered that vascular endothelial growth factor α (VEGF-α), is secreted by a specific subset of type I conventional dendritic cells (cDC1s) during infection with either *C. acnes* or *S. aureus* in the mouse model of inflammatory acne. This secreted VEGF-α has the ability to attract neutrophils to the site of infection (97).

Chemokines are another important factor in the immune response. They act as critical mediators of immune cell migration during immune surveillance and immune development (98). In acne vulgaris, keratinocyte-secreted chemokines, including CCL2 and CCL5, TREM2^+^ macrophage-secreted chemokines, such as CXCL16 and SPP1 (37), and sebocyte-secreted chemokines, like CXCL8 (13), have the ability to attract immune cells to the acne region.

## 4 Adaptive immune response

**Figure 2:**
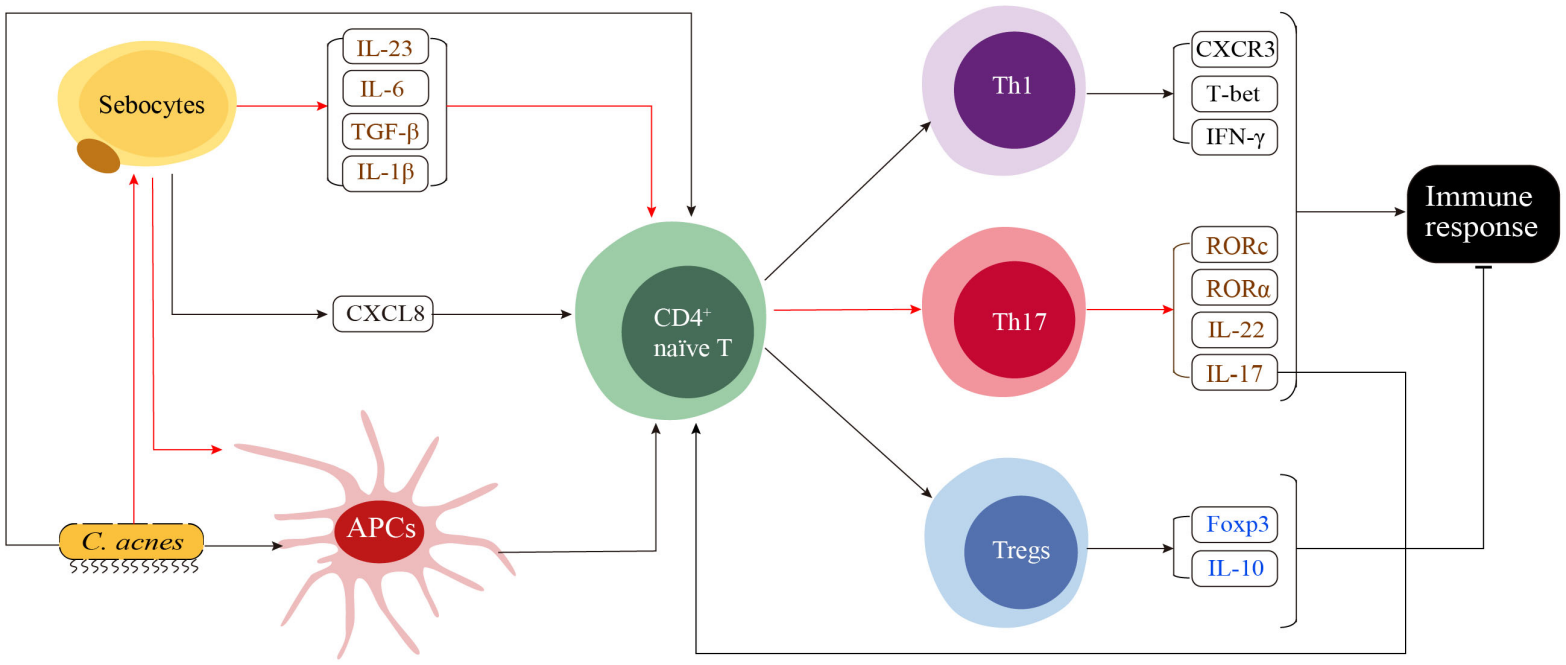
A proposed model of the adaptive immune response in acne. The innate immune response stimulates skin cells, such as sebocytes, to secrete CXCL8, which in turn recruits CD4^+^ naïve T cells to the sites surrounding the pilosebaceous unit. CD4^+^ naïve T cells in this region receive various stimulating signals, including cytokine signals from sebocytes, pathogen stress signals from *C. acnes* and major histocompatibility complex signals from *C. ances* and seboctes-stimulated antigen presenting cells (APCs). These signals determine the differentiation of CD4^+^ naïve T cells into Th1, Th17 or Tregs. *C. acnes* directly induces the differentiation of CD4^+^ naïve T cells into both Th1 and Th17 cells. Th17-related cytokines secreted by sebocytes, including IL-6, TGF-β and IL-1β, as well as the IL-23 secreted by currently unidentified cells, induce the differentiation of CD4^+^ naïve T cells toward Th17. Additionally, the functional interaction between sebocytes and *C. acnes* induces the maturation of APCs, resulting in a preferential generation of Th17 cells over Th1 cells. The activation of Th1 and Th17 cells enhances both the innate and adaptive immune responses, while activated Tregs function as suppressors that negatively regulate the immune response.

Theoretically, adaptive immunity is activated upon exposure to antigens presented by the major histocompatibility complex (MHC) of antigen-presenting cells. The adaptive immune response is characterized by a slow speed, high specificity, and the ability to develop memory. Dendritic cells (DCs) are the key professional antigen-presenting cells activating T and B lymphocytes (3). However, specific DC subtypes responsible for the antigen presentation in acne remains understudied. In this section, we illustrate the immune cells that have been identified as critical players in adaptive immune response. And a model proposed based on these discoveries is presented in **Figure 2**.

Th1 and Th17 represent distinct subsets of CD3^+^ CD4^+^ T helper cells and are the predominant immune cell populations infiltrating the dermal papilla and around the perifollicular regions in early-stage acne. Th1 cells are recruited and activated in early acne lesions, as determined by Mouser et al. (99). who generated 14 T-cell lines from early papular inflammatory acne lesions with enhanced proliferative responses to antigens derived from *C. acnes*. Further, a Th1 cytokine pattern characterized by high IFN-γ production and low IL-4 production indicated the involvement of Th1 cells in the adaptive immune response in acne vulgaris, particularly in the early stage (99). This result was later verified by Agak et al. in a study of peripheral blood mononuclear cells treated with *C. acnes* (9).

The effector functions of Th17 cells differ from those of Th1 cells (99, 100). The differentiation and proliferation of Th17 cells are facilitated by various cytokines, such as IL-17, IL-1β, IL-6, TGF-β, and IL-23 (9). Agak et al. (9) found that Th17 can be differentiated from CD4^+^ T cells rather than CD8^+^ T cells when stimulated by *C. acnes*. This differentiation was indicated by the upregulation of IL-17 related genes, including IL-17, IL-17 receptor genes (IL-17RA and IL-17RC), and the downstream transcription factors (RORα and RORc). Moreover, Vitamin A (ATRA) and D (1,25D3), two commonly used immunomodulators in acne therapeutics, can inhibit *C. acnes*-induced Th17 differentiation (9).

Matti et al. demonstrated an accumulation of CD4^+^ IL-17^+^ cells in close proximity to the PSU, which suggests the interaction between sebocytes and these CD4^+^IL-17^+^ cells (13). Moreover, the chemoattractant process can be further enhanced by proinflammatory cytokines, such as IFN-γ, IL-17, and TNF-α. Although the CD4^+^ CD45RO^+^ effector T cell subset was the most abundant T cell subset attracted by sebocytes, functional activation was not observed. In contrast, the small number of CD4^+^CDRA^+^ naïve T cells attracted by sebocytes are targets for differentiation into Th17 cells. This differentiation is mediated by the sebocyte supernatant in a manner dependent on IL-1β, IL-6, and TGF-β as well as by the DCs generated in the presence of the SZ95 supernatant. This population of activated Th17 cells is characterized by elevated levels of IL-17 and IL-22. The supernatant of *C. acnes*-prestimulated SZ95 sebocytes has the potential to influence the primary capacity of DCs, leading to increased differentiation of naïve T cells toward Th17 rather than Th1 subsets (13).

T regulatory cells (Tregs) is characterized by high expression levels of IL-10 and FOXP3. IL-10 is an anti-inflammatory cytokine, and FOXP3 plays as a suppressive role in the immune system. Elevated expression levels of these molecules were observed in both the serum and papillary dermis of patients with acne. This finding suggests that Tregs cells may contribute to the prevention of autoimmunity and the suppression of excessive immune response in acne (7).

## 5 Sequential involvement of immune cells

**Figure 3:**
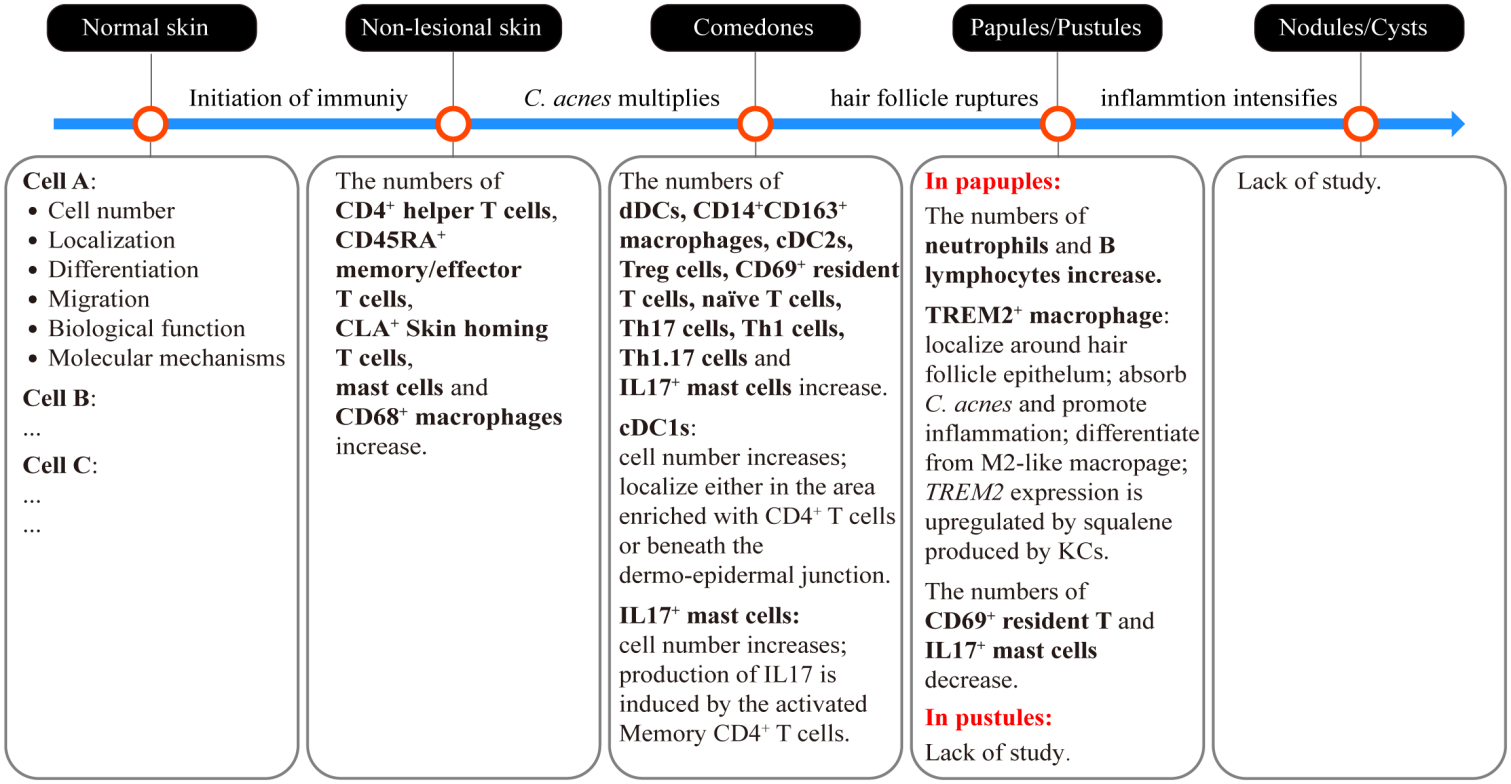
Sequential involvement of immune cells in different types of acne lesions and the critical events that drive the initiation and progression of acne. The dysbiosis of microorganisms, sebum, neuroactivity, or environmental virulence factors triggers the initiation of an immune response. At this stage, immune cells have already infiltrated the skin without any visible clinical lesions (non-lesional skin). This early-stage immune activity induces the hypercornification of the hair infundibulum and overstimulates sebocyte function, resulting in the formation of microcomedones and later comedones. *C. acnes* multiplies during the development of comedones. Continued stress from *C. acnes* and the enlargement of comedones on the hair follicles result in the rupture of the follicle wall. The contents of the comedones, including microorganisms, sebum and keratin squamae are released into the dermis, leading to the formation of papules or pustules. Ultimately, if the inflammation intensifies without control, papules or pustules may develop into more severe lesions, such as nodules and cysts. The sequential involvement of immune cells currently observed in different types of acne lesions is summarized in the corresponding text box. Further studies are needed to obtain more information, including the cell numbers, tissue localization, differentiation and migration trajectory, as well as the biological functions of specific immune cells involved in different acne lesions. Moreover, it is crucial to elucidate the molecular mechanisms that underlie these characteristics to facilitate the development of targeted treatment and prevention strategies for acne.

In addition to characterizing the detailed functions of the aforementioned immune-associated factors, it is crucial to elucidate the order in which immune-related cells participate in acne development. Recently, Eliasse et al. used a multipronged approach that included flow cytometry, confocal microscopy, and bioinformatics to demonstrate that distinct cell populations play dominant roles at different stages of acne development (8). Combined with the findings of *in vivo* studies of inflammatory processes (8, 96, 101–103), a primary immune landscape of the acne process is beginning to emerge (**Figures 2**, **3**). Disrupted homeostasis caused by the dysbiosis of microorganisms, sebum, neuroactivity, or environmental virulence factors triggers a response in the cells of PSUs. The initial immune response is triggered by keratinocytes and sebocytes, that secrete AMPs, cytokines and chemokines to attract immune cells, including CD4^+^ helper T cells, CD45RA^+^ memory/effector T cells, CLA^+^ skin homing T cells, mast cells and CD68^+^ macrophages. Although with immune cells infiltration, there are no clinical symptoms at this stage (non-lesional skin). Infiltrated immune cells in the comedone lesions include APCs (CD14^+^ dermal DCs, CD14^+^CD163^+^macrophages, CD1c^+^ conventional DC2s, conventional DC1s), CD3^+^CD4^+^ helper T subsets (CD69^+^ resident T cells, regulatory T cells, naïve T cells, CD161^+^CXCR3^-^ Th17 cells, CD161^-^CXCR3^+^ Th1 cells, CD161^+^CXCR3^+^ Th1.17 cells) and IL17^+^ mast cells. These cells are predominantly cluster in the papillary dermal, periductal or perivascular regions. As the comedones progress into papules and pustules, the number of CD69^+^ resident T cells decreases, while neutrophils and B lymphocytes start to be recruited in large numbers within the lumen of pilosebaceous ducts (**Figure 3**).

However, little is known about the immune processes at the stages of pustules, nodules, cysts, and scars, and there are inconsistencies in study results (**Figure 3**). For instance, Demina et al. (104) revealed a decrease in anti-inflammatory cytokines (IL-4 and IL-10) in the serum of patients with acne. This suggests that an insufficient anti-inflammatory immune response may contribute to immunodeficiency. However, Kelhala et al. (96) found higher levels of IL-10 and Foxp3^+^ Tregs, which can prevent autoimmunity and suppress the immune response in acne. Further investigations focused on various stages of acne are required to provide additional clarification regarding these contradictory findings and to construct a more comprehensive immune response process map at a more precise temporal-spatial scale.

Recently, single-cell sequencing technology has emerged as a powerful tool for acne research (37, 105). In an impressive example, Do et al. (2020) used single-cell and spatial RNA sequencing techniques to successfully identify a distinct subcluster of macrophages, known as TREM2^+^ macrophages. These macrophages exhibit specificity and accumulate in early-stage acne lesions. Differentially expressed genes in TREM2^+^ macrophages are involved in lipid metabolism and proinflammatory processes. A pseudo-time analysis revealed that TREM2^+^ macrophages were differentiated from M2-like macrophages. Spatial RNA sequencing and ultra-high-resolution Seq-Scope have shown that TREM2 is localized in proximity to the hair follicle epithelium, which expresses squalene epoxidase. Wet experiments demonstrated that keratinocytes present in acne lesions exhibit an increased capacity for squalene synthesis. Squalene stimulates TREM2 expression in macrophages. Increased TREM2 expression enhances the phagocytic capacity of macrophages, allowing them to effectively absorb *C. acnes* and lipids. However, absorbed squalene inhibits the oxidative killing of *C. acnes*. Additionally, the upregulation of 25 proinflammatory genes was associated with the recruitment and activation of immune cells (37). These data provide a basis for identifying the specific cell types involved in the development of acne and provide information on their distribution, differentiation and migration trajectory, gene expression pattern, and biological function as well as interrelationships between different skin cells and microorganisms (**Figure 3**).

## 6 Conclusion

There has been substantial progress in our understanding of the mechanisms underlying the immune responses associated with acne development. The immune response in acne is intricately connected to the modified profiles of *C. acnes* phylotypes, related gene pools, and altered transcriptional activity. It is crucial to recognize that acne is not solely determined by the quantities of secreted sebum but also by the composition of diverse lipid species and the oxidant/antioxidant and saturated/unsaturated ratios, which ultimately determine whether these lipids exert beneficial or detrimental effects. The recognition of microbial pathogens and lipid mediators is attributed to TLRs, PAR-2, and CD36. The NF-κB, AP-1, and NLRP3 inflammasome signaling pathways play crucial roles in the expression and secretion of soluble factors associated with immune-inflammation. Conversely, the PPAR and HDAC8/9 pathways are responsible for the negative regulation of these immune-and inflammation-related soluble factors. Inflammatory events precede hyperproliferative alterations in keratinocytes within the pilosebaceous duct. Single-cell and spatial multi-omics techniques have provided key insights into the distribution, expression patterns, and functional characteristics of specific skin immune-associated cells in the context of acne and are important tools for further research.

Additional research is necessary to fully understand the influence of particular microbial phylotypes, genetic factors, lipid species compositions, and neuroendocrine activity on immune responses linked to acne *in vivo*. Obtaining comprehensive data is crucial to accurately portray immune activity in diverse lesion types with varying degrees of acne severity. Furthermore, it is imperative to determine whether these immune processes differ according to individual genetics, living conditions, and lifestyle choices. A thorough understanding of the immune processes involved in the development of acne can facilitate the implementation and advancement of targeted treatments and prevention approaches.

## Author contributions

ZJ: Conceptualization, Writing – original draft, Writing – review & editing. YS: Writing – review & editing. LH: Conceptualization, Supervision, Writing – review & editing.
